# Pharmaceutical mobile application for visually-impaired people in Thailand: development and implementation

**DOI:** 10.1186/s12911-021-01573-z

**Published:** 2021-07-16

**Authors:** Acrapol Nimmolrat, Pattaraporn Khuwuthyakorn, Purida Wientong, Orawit Thinnukool

**Affiliations:** 1 College of Arts, Media and Technology, Chiang Mai University, Chiang Mai, 50200 Thailand; 2 Department of Pharmaceutical Care, Faculty of Pharmacy, Chiang Mai University, Chiang Mai, 50200 Thailand; 3 Research Group of Embedded Systems and Mobile Application in Health Science, College of Arts, Media and Technology, Chiang Mai University, Chiang Mai, 50200 Thailand

**Keywords:** Pharmaceutical applications, Visually-impaired, Usability design

## Abstract

**Background:**

Most mobile pharmaceutical applications produced for people with visual disabilities in Thailand fail to meet the required standard due to poor-quality regulations, defective design, lack of user support and impracticality; as a result, visually-impaired people are unable to use them. This research is motivated by the limited use of this technology in primary medical services and its aim is to enable people with disabilities to access effective digital health information. The research objective is to analyse, design and develop a mobile pharmaceutical application with functions that are appropriate for visually-impaired users, and test its usability.

**Results:**

Based on the design and development of the application, it contained five necessary functions. When testing the usability and users’ satisfaction, it was found that the input or fill of information in the application was of low usability. According to the test results, the medicinal database function was missing 71 times and the voice command function was missing 34 times. Based on users’ satisfaction results, users who had the highest level of usage gave higher average scores to users’ attitude, users’ confidence, user interface and system performance than those with lower levels of usage. The scores of both groups were found to be the same when discussing the implementation of the development.

**Conclusions:**

This mobile application, which was developed based on the use of smart technology, will play an important role in supporting visually-impaired people in Thailand by enhancing the efficacy of self-care. The design and development of the application will ensure the suitability of many functions for visually-impaired users. However, despite the high functional capacity of the application, the gap in healthcare services between the general public and disabled groups will still exist if users have inadequate IT skills.

**Supplementary Information:**

The online version contains supplementary material available at 10.1186/s12911-021-01573-z.

## Background

The use of technological innovation in the field of healthcare continues to improve the quality of human life. Since smart devices are increasingly common and accessible to everyone everwhere [1, 2], there are many types of smart applications and information systems that can help patients in several ways, such as assisting their rehabilitation, monitoring and tracing their health condition, providing a consultancy service and suggestions in relation to their medication [3–10].

Smart technology, especially in the form of mobile applications, provides users with a great many benefits, including reducing the time and cost of treatment and making it convenient for them to find health-related information. These apps have become more accessible and helpful to many users, including those who are visually-impaired [11]. For these reasons, the Thai Government has introduced a policy that involves the application of smart technology in healthcare services in all parts of Thai society [12, 13]. This is not only beneficial for the general population who use the Thai national healthcare system, but it also supports the needs of people with disabilities, which reduces social inequality. However, although this policy is based on good intentions and a strategy that enables people with disabilities to access digital information, the requisite technology is often inaccessible or only partially accessible to them. Most medical services or tools in Thailand are dedicated to general users rather than those with impaired vision due to the eHealth Strategy of the Ministry of Public Health (2017–2026) of Thailand [14].

According to the 2017 annual report of the Ministry of Social Development and Human Security on disability, approximately 3.08% of the total population of Thailand is disabled, and 9.99% of them or about 200,000 people have impaired vision [15]. Therefore, the gap in the healthcare service between the general public and disabled groups needs to be closed to improve social equality, and smart technology is the key to closing that gap. Various research institutes and organisations in Thailand have developed applications to assist people with disabilities, and there is a strong demand for the use of smart technology, especially in mobile devices [16].

Several functions of currently-available mobile applications have been designed based on high capacity and high performance, which is beneficial for the majority of users. However, few applications on smartphones have been specifically designed with functions that benefit people with impaired vision or have vision disability modes. Despite evidence of the existence of more than a thousand commercial health applications [11], there are limited mobile applications for users who are visually-impaired, and those that are available fail to meet the required standard of quality, design and accessibility[17–19].

Other problems are users’ level of IT literacy and their ability to access smart devices [20]. A low level of IT literacy reduces their ability to use a smart system, as well as understand and access up-to-date information. Despite the recent evolution of various methods and tools to access information on smart systems, there have been no explicit attempts to develop or design an electronic health application to serve those with impaired vision [21, 22]. Therefore, the purpose of this study is to design and produce a mobile health application that gives all people, especially those who are visually-impaired, an equal opportunity to access health information in order to eliminate inequality and enhance the social stability in Thailand.

Two research questions need to be answered to resolve the aforementioned problems. The first is how to develop and design an appropriate practical mobile application to enable visually-impaired users to access primary self-help when they are sick. The second is what an appropriate user interface design and functional system for visually-impaired users should be. The application in this study is based on evidential research of an appropriate user interface and functionality. The aim is to analyse, design and implement a mobile pharmaceutical application called “Ru Tan Ya” in the Thai language, which enables users to manage their medication and can be applied to both iOS and Android systems. A user-centred process is utilised, with a focus on practicality with good usability [23, 24] based on Shneiderman's Eight Golden Rules of interface design, Nielsen’s Ten Heuristics and human–computer interaction (HCI).

A group of visually-impaired members of the Vision Disability Association in northern Thailand were recruited to trial and evaluate the system based on three research objectives: (1) To identify the problems experienced by visually-impaired users who attempt to use smart technology to access primary self-treatment when they are sick; (2) To analyse, design and develop a mobile pharmaceutical application with a user interface and functionality appropriate for those who are visually-impaired; and (3) To test the usability of the application and implement it. The outcome of this research is expected to assist visually-impaired people, who find mobile applications more challenging to use than other disabled groups do.

## Literature review

### User-centred process

This is a developmental research that emphasises the use of an analysis, design and development method to produce a mobile application that can meet target users’ needs; hence, a user-centred process framework of an iterative design was applied. Since this required the developer or designer to focus on the needs of specific users, the design details included usability, users’ characteristics, usage environment, tasks, workflow of the system, and related products or services. A great many researchers in the healthcare and medicinal services fields have used this process framework to design and develop their systems [25–27] and, in this case, a well-formed interview process enabled the researcher to focus on understanding the target users’ healthcare needs and related problems [28]. The software was developed based on a user-centred approach that consisted of 5 steps: (1) identifying users’ problems and needs; (2) analysing users’ problems and needs; (3) designing the user interface; (4) developing the application, and (5) testing its usability.

### Usability design for the visually-impaired

Designing a system that can be used by people with visual disabilities requires human–computer interaction (HCI). This involves mobile computing with a focus on the interface between the user and computer [29]. Therefore, the guidelines of HCI, including Shneiderman’s Eight Golden Rules, Norman’s Seven Principles and Nielsen's Ten Heuristic Principles, were utilised for the design in this study.

Although the above-mentioned methodology is not specifically focused on accessibility design, conceptual design principles, such as the provision of informative feedback, designing for error, simplifying the task structure design and recognition, were also adopted based on the studies of several researchers, as shown in Additional file 1.

Lessons learned from practicing the design elements of health applications were used [30–33] to design the system’s usability, together with the HCI guidelines and the key points of the design are detailed below.

I. Touchscreen accessibility: Users with visual disabilities need to use a touchscreen to access the application; hence, the design must be focused on the proportion of the screen, since different screen sizes provide different levels of convenience. Accessibility can be increased by enlarging the text size, which enables users to easily assess the correct position on the screen, and alternative image text can be used to describe images [34].
II. Voice user interface: This feature is designed to increase accessibility by means of interactive communication between the user and the system. This suggests that the designer should consider the system’s messages, grammar and speed of conversation, and only include relevant content and true statements [33, 35].
III. Slide rule and navigator techniques: These are entirely speech-based (sound display) and have no visual representation. The user interacts with the system by touching the screen. The number of touches is based on the user’s decision, such as one touch refers to forward and two touches refer to backward [32, 36]. The positioning of the content on the screen, such as header, menus, content and footer, is subject to the appropriate structure [25].

### Application testing

Since the design of the user interface in this research was based on Shneiderman’s Eight Golden Rules of interface design and Nielsen’s Ten Heuristics, the principle design of the human-centred theory was used for testing purposes. In addition to applying heuristics to test the usability of the proposed system, it was tested by a traditional questionnaire because a combination of heuristics and non-interactive methods, such as interviews, focus groups and task completion, has been found to be an effective method for testing usability [28]. Hence, when trialling the proposed application, the usability test consisted of two steps focused on users based on lessons learned in practice.

The criteria of the design elements used to trial the usability of the health application in this research [32–36] are defined and evaluated in Table 1.

**Table 1:** Definition of the evaluation of the criteria of the Ru Tan Ya application

Criterion	Definition of evaluation
Occurrence of issues	User cannot access or utilise one of the following functions; Exit or return to the previous menu Type in the textbox Use voice recognition Incompatible voice recognition Finger sliding of voiceover touch in the wrong position > 3 times
Bugs	User encounters bugs or gets stuck trying to use certain functions
Obscure visual representation	User fails to understand voiceover screen, or slides finger in the same position more than 3 times and restarts a task more than twice
Obscure operation	One of the following issues occurs when operating the application; User slides finger into the same position more than 3 times User closes the screen and restarts a task, but does not complete it User cannot use a certain function User cannot type in the textbox
Missing information	User is confused by the information and does not understand the content or voice display menu
Misunderstanding	User is confused by the information and navigates to the wrong task User cannot use certain functions and is unable to finish a task. User inputs incorrect typing to the textbox and touches on the wrong task

The second step consisted of after-use interviews, in which users were asked to answer four sets of questions related to the use of the application. The first 8 questions were related to users’ attitude toward the design of the system, while the next 10 concerned their confidence in using it. The subsequent 7 were related to the usability of the user interface, while the last 9 concerned the system’s performance. The list of questions can be found in Table 6.

## Research methodology

### Procedure

This Ru Tan Ya mobile application is being designed and developed due to the need to solve the issue of inappropriately-designed applications for visually-impaired users. Many researchers in the healthcare and medicinal services fields have designed and developed their systems based on a user-centred approach [26, 27, 37]. The procedures in this study, which had human participants, fully complied with the ethical standards of the institutional and/or national research committee. Therefore, the approach used to develop the software in this research consisted of the 5 steps described below.

**Step 1:** Identification of users’ problems and needs. This step involved the collection and analysis of essential information by interviewing visually-impaired volunteers with a focus on identifying the functions that are necessary for visually-impaired people to access pharmaceutical medication using a digital device. A well-formed interview process enabled the researcher to thoroughly understand users’ problems and needs related to healthcare [28].

**Step 2:** Analysis of problems and needs. An analysis of users’ problems and needs plays a vital role in developing an application. In this case, the results of step 1 facilitated a consideration of the application’s ability to enable users with impaired vision to conveniently access pharmacies and medicinal support, while the results of this step provided a blueprint of a function analysis and summary design.

**Step 3:** Application design. This step was based on the results of the previous step. It involved the design of a user interface based on an analysis and review of the literature of design for the visually-impaired in order to create an application that would perform well on smartphones with a disability mode. After completing the analysis of the user interface design, a wireframe design was created for a summary of the screen elements of the application, such as the navigation components, input and output controls, screen proportion design, and a menu list navigation to drag and drop elements on the screen. Moreover, the usability was considered based on techniques found in the literature review.

**Step 4:** Application development. An Ionic Framework was selected to develop the application in this step. This is a tool for developing cross-platform applications from a single code base for both native iOS and Android operating systems. The real-time database, Firebase, which includes public and private medicinal information, users’ data and a list of medicinal categories, was used to manage the data for the proposed Ru Tan Ya pharmaceutical application.

**Step 5:** Application testing. After the application had been developed, its usability was tested in order to evaluate users’ attitude toward its practical use and ability to maintain their confidential information. As mentioned earlier, the application’s usability was assessed based on the principle design in the literature review, and it was tested twice, as shown below.

1. Each function was subjected to a usability test by volunteers, who used the application in a real-life scenario that included the following steps: (1) finding information about medicine for diarrhoea; (2) after finding it, setting an adherence timer; (3) finding at least one pharmacy; (4) finding previous medication history; and (5) recording medicinal information on the application.

Thirty samples were selected and the usability test was observed until the scenario ended. Each sample took approximately 10–20 min to complete the five steps without any help from the researcher. The voice-over touch mode or disability mode was required to be enabled on the smart device to use with the application.

2. The visually-impaired volunteers provided usage feedback after trialling the application based on the questionnaire shown in Tables 7–8, which contained a list of questions related to using the application for inclusion in the users’ interviews. The visually-impaired volunteers were asked to rank their response to each question in the questionnaire on a ranking scale after the researcher had read it to them twice.

## Participants

### Patients and public involvement

No patients were involved in this research.

### Demographics

Sixty volunteers (n = 60) from the 251 active members of the Vision Disability Association in northern Thailand were recruited based on a simple random sampling technique. The inclusion criteria were that they must be members of the Vision Disability Association, own a smartphone, be more than 90% vision-impaired and be willing to take part in the research, while the exclusion criteria were feeling uncomfortable with being interviewed and not possessing a smartphone. The invitation was announced to all members by the staff of the Vision Disability Association. The names of the participants were provided as a result of the sampling, and they were invited to join the research based on documentary ethical clearance and approval. The demographic details of the samples, including the characteristic of their visual disability, are shown in Table 2.

**Table 2:** Demographics of the visually-impaired volunteers (n = 60)

Category	Characteristic	Percentage (n)
Gender	Male	53.33 (32)
	Female	46.67 (28)
Vision condition	Low vision	6.67 (4)
	Blindness	93.33 (56)
IT literacy skills	No smartphone experience 1	13.33 (8)
	Beginner smartphone user 2	25.00 (15)
	Intermediate smartphone user 3	58.33 (35)
	High-level smartphone user 4	3.33 (2)

### Data collection and analysis

The data was collected from the participants in two stages, the first of which involved collecting the data from step 1 to identify users’ problems and needs. The visually-impaired volunteers were separated into groups of ten for a 30-min interview based on the four open-ended questions shown in Table 3. The results were analysed according to the frequency of each group’s answers.

**Table 3:** Results of interviews of visually-impaired interviewees using open-ended questions based on the most frequent answer (cut off at 10)

Questions	Group Answers	Frequency
1. How do you find medicine when you are sick?	Ask friend or relative to buy medicine at pharmacy or hospital	42^(1)^
	Use Google and voice recognition mode on smartphone	35^(2)^
	Go to pharmacy or hospital myself	28^(3)^
	Use old prescribed medicine or home remedy	19
	Find treatment information via website using a computer	12
2. Is it easy for visually-impaired people to use smart tools to find medicine? What makes it difficult for you to access the required medication?	Not easy. It is difficult to use smart tools	43^(1)^
	Not easy. Websites delivering information with pictures are inaccessible	42^(1)^
	Not easy. Voice recognition mode from Google translates incorrectly and users are not familiar with medical terms	32^(1)^
	Difficult because I am not convinced by some data on the web	26
	Not all the Google search results can be easily validated	19
	Most Google search results are presented in English on foreign websites	16
	Medicine information is different on each website	12
	etc	9
3. Why do visually-impaired people not use pharmaceutical mobile applications on a smartphone?	Difficult to use	48^(1)^
	Pharmaceutical information on applications hard to understand	26^(2)^
	My device has a low performance	23^(3)^
	The application does not support a disability mode	14
	Not a smartphone owner/can’t afford it	12
	Didn’t know about it	8
4. What would be a good solution for a future pharmaceutical mobile application?	A function that helps to find medicinal information easily and provides the appropriate information for primary treatment	42^(1)^
	A function that explains self-preparation when sick	39^(2)^
	A function that navigates directly to the pharmacy, clinic or hospital with just one click	37^(3)^
	A function that explains the treatment steps and medicine administration	37^(1)^
	A function that records treatment information	35
	A doctor appointment-reminder function	35
	An application with disability mode support	34
	etc	9

(1) (2) (3) are rankings

The second stage involved collecting the data from step 5 of the application test from the same sample to examine users’ satisfaction, attitude toward and usage of the application from the questionnaire. After trialling the application, the questionnaire was modified based on Thinnukool [38], as shown in Table 6. The interview questions were divided into four parts: (1) users’ attitude toward the use of mobile applications as a tool for primary care treatment; (2) users’ confidence in using this tool; (3) users’ evaluation of the user interface design; and (4) feedback about the performance of the application’s functionality. The results were analysed by comparing the average score of the response to each question. Moreover, the observation from trialling the application indicated that the usability test had highlighted the absence of five points. The results were analysed based on frequency.

## Results

### Results of identifying users’ problems and needs

This part was related to understanding these visually-impaired users’ problems with the application in order to design a system that is appropriate for them. The answers and comments of the visually-impaired volunteers from the Vision Disability Association are summarised in Table 2. They were asked to highlight the problems they had encountered between the 10^th^ May and 10^th^ June, 2018, based on open-ended questions.

### Results of analysis of problems and needs

The purpose of this step of the application development was to understand users’ needs, problems and limitations/conditions based on their experience. An analysis of these problems and needs provided the necessary data to design the Ru Tan Ya application. Its functions were considered stepwise, as shown in Table 4.

**Table 4:** Functional analysis and design of the Ru Tan Ya mobile application

Problem	Solution	Benefits	Design point
Difficult to find medicinal information	Provide a function to access accurate medicinal information	User can access proper medicinal information	Medicinal information to support user on a smart device platform
Difficult to find information on website or application. Difficult to navigate to drug stores	Provide a suitable and compatible function for user device and direct mapping direction	Practical use for visual disability users by providing convenient and easy access to drug information	Adequate information with a directional map on the application
Language barrier	Use the Thai language in the application	Clear understanding	Thai language
Overload of information about a particular drug	Limit the amount of information appearing in the application	Precise and easily understandable information is provided	Display adequate information
Incorrect Google translation/ users’ lack of knowledge of medical terms	Simplify drug information classification	Drug information database is easy for all users to navigate	Function easily navigated by voice recognition input
Application does not support disability mode/ low capacity and performance devices	Design element to support touching Application runs smoothly on a device with a low capacity of resources	Easily understandable information input/output. Application can be used on low-performance device	Adapt developmental technique

In response to the research question of what functions an application designed especially for visually-impaired users should have, Table 5 indicates the design of the application based on users’ problems, the way to solve them and the estimated benefits. Although most problems have arisen due to users’ lack of previous experience of information systems, some of them have been caused by issues with the applications themselves and insufficient IT literacy. Therefore, the aim of this research is to develop an application that is suitable for use by the visually-impaired population in Thailand based on an analysis of the functionalities and design shown in Table 4.

**Table 5:** Analysis of the functionalities and design of the Ru Tan Ya mobile application

Functionality	Design	Usage method	Results
Searching for medicinal information	Searching via voice recognition Interface presented in Thai Support disability mode Minimal design Flexible and efficient use Matching system with the real world Touch screen Necessary elements on screen Prevent errors Offer informative feedback Consistency of elements	User slides and holds the button to use voice recognition Barcode scanner is available to input data from medicine container	Appropriate information such as name of drug, properties, method of administration, admixture, drug group, method of consumption in case of missing doses, and preservation
Reminder	Notification to take medicine Minimal design Touch screen Prevent errors Offer informative feedback Consistency of elements	User slides to function and slides to add drug list, then sets reminder	Notification to remind user on main screen of smartphone
In-app drugstore direction map	User can find drugstore based on the fastest route Direction must be based on tactile paving for visually-impaired people (different from Google map) Minimal design Flexible and efficient to use Free control for users Touch screen Necessary elements on screen Prevent errors Consistency of elements	Drug list and Drugstore location will be prompted in just one click	Map will suggest drugstore locations
Medication history	List of previously-used medication including course details Editable Disability mode compatible Touch screen Necessary elements on screen Offer informative feedback Consistency of elements	One-click history information edit or remove User opens and clicks hold over list	List of information displayed on touching voiceover
Customised drug database	More drug information can be added manually by user or pharmacist Aesthetic and minimalist design Free control for users Visibility of system status Touch screen Necessary elements on screen Help, diagnosis and recovery from errors	User slides and holds the button to use voice recognition to find or record new drug list on database	New drug information added to mobile memory

### Results of application design

**Fig. 1:**
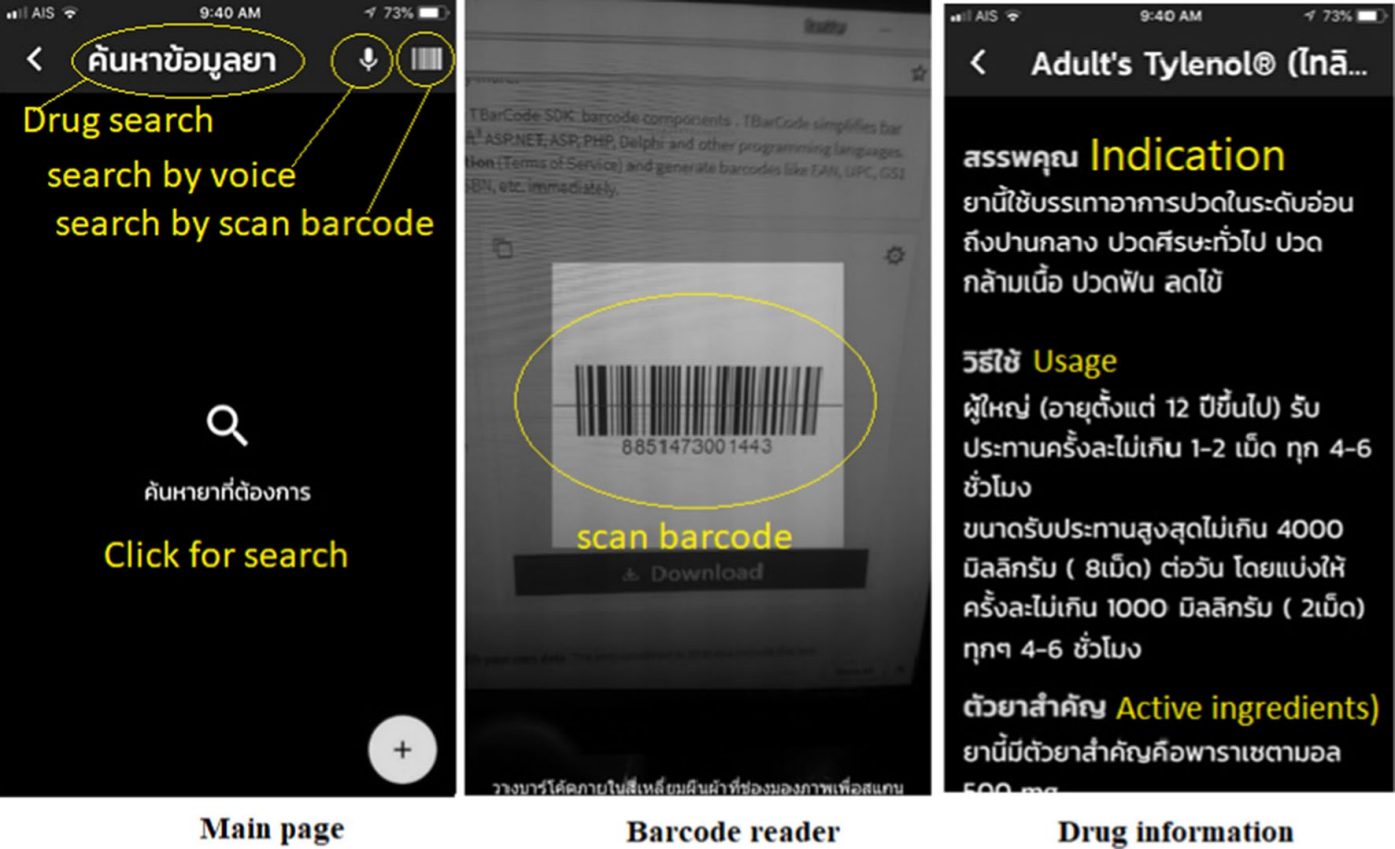
Searching for medicinal information. Purpose: To search for and access medicinal information using voice recognition and barcodes, which are tools to input the name of a medicine or symptom; meanwhile, a barcode may be a suitable tool in cases where users need information about an unknown drug

**Fig. 2:**
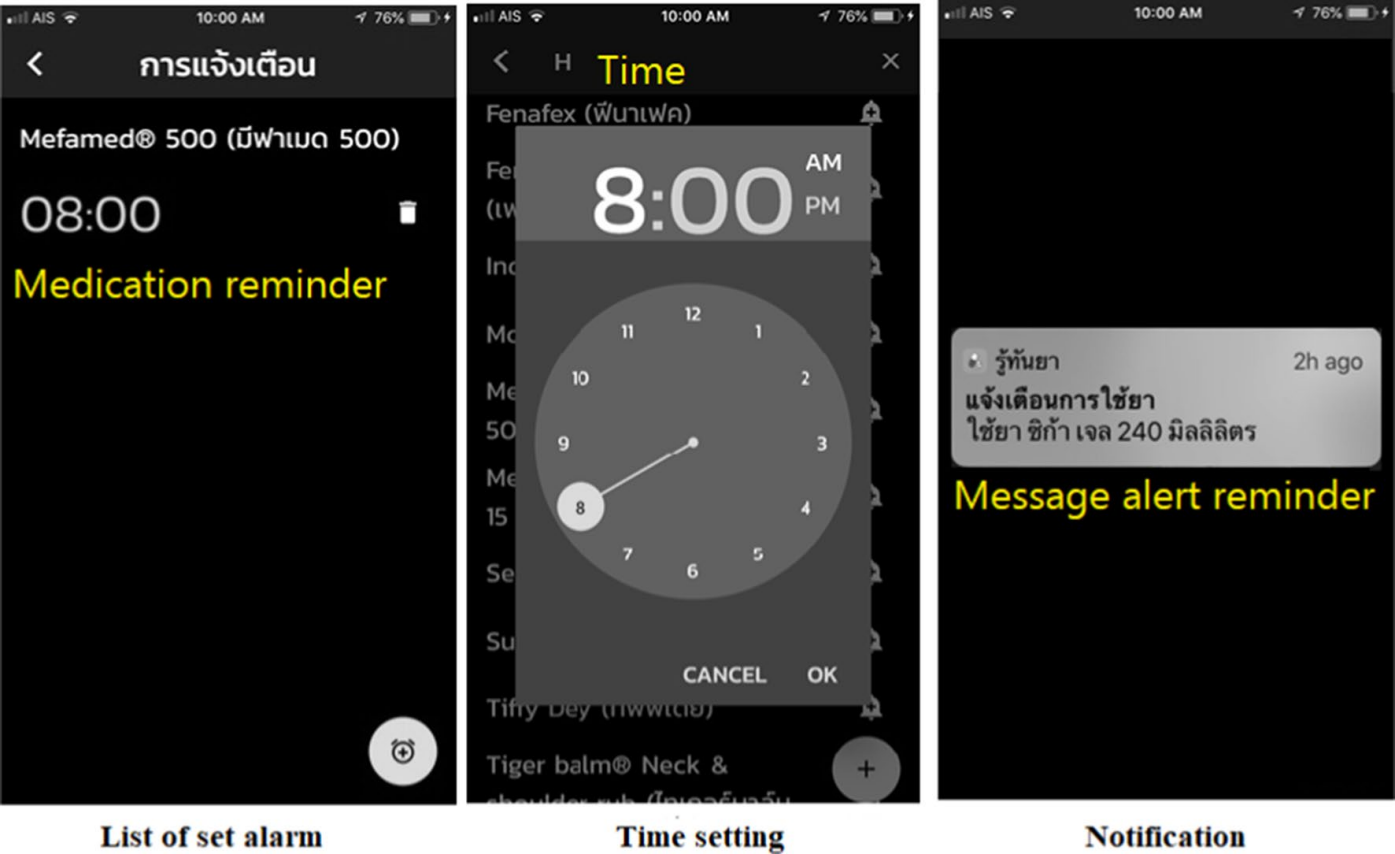
Reminder function to improve medication adherence. Purpose: A reminder function that notifies users to take medication at the appropriate time to optimise medication adherence

**Fig. 3:**
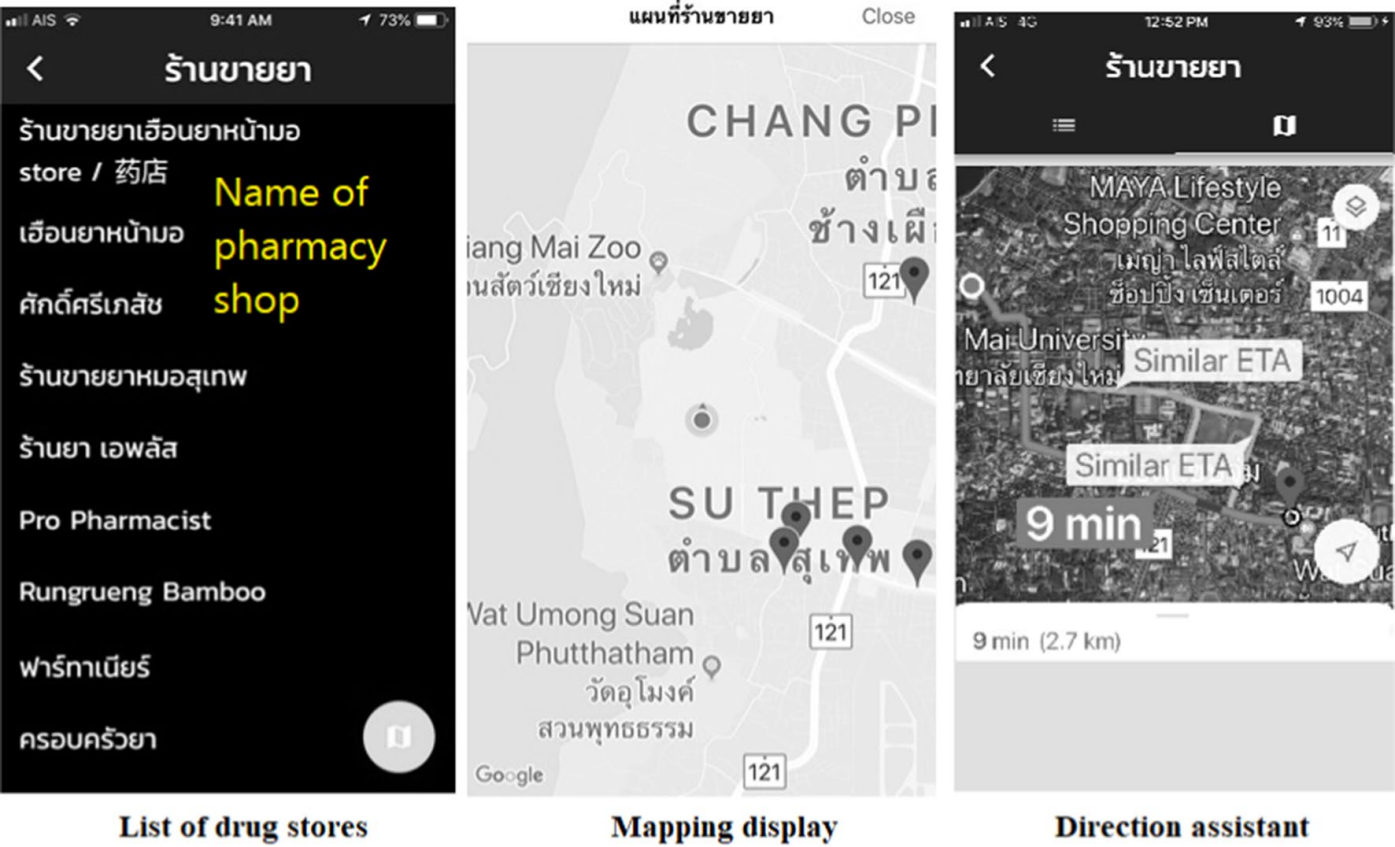
Map of direction to drug stores. Purpose: To help users to search for local pharmacies and find a map to the nearest one

**Fig. 4:**
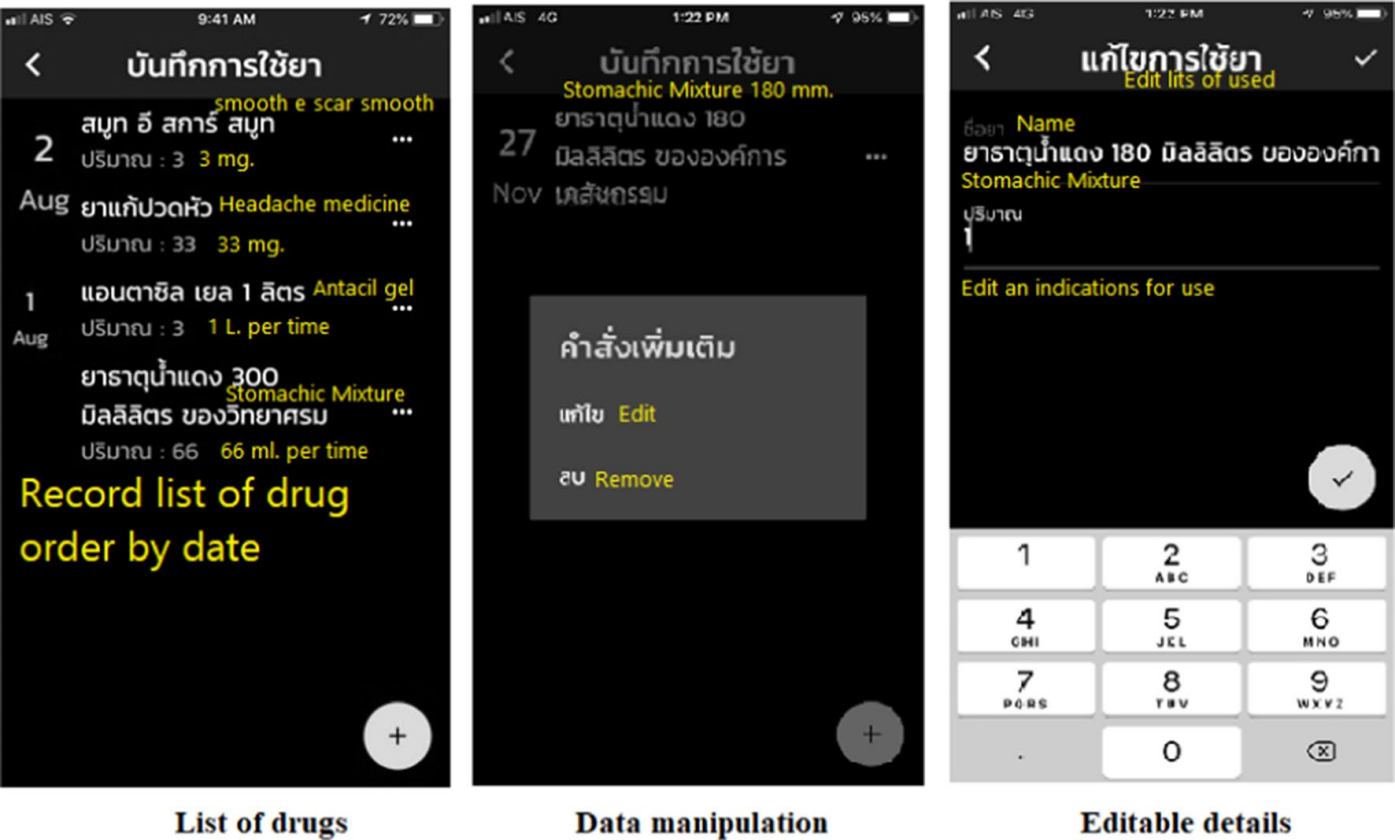
Medication history function. Purpose: To record a log of individuals' medication history for further treatment or monitoring

**Fig. 5:**
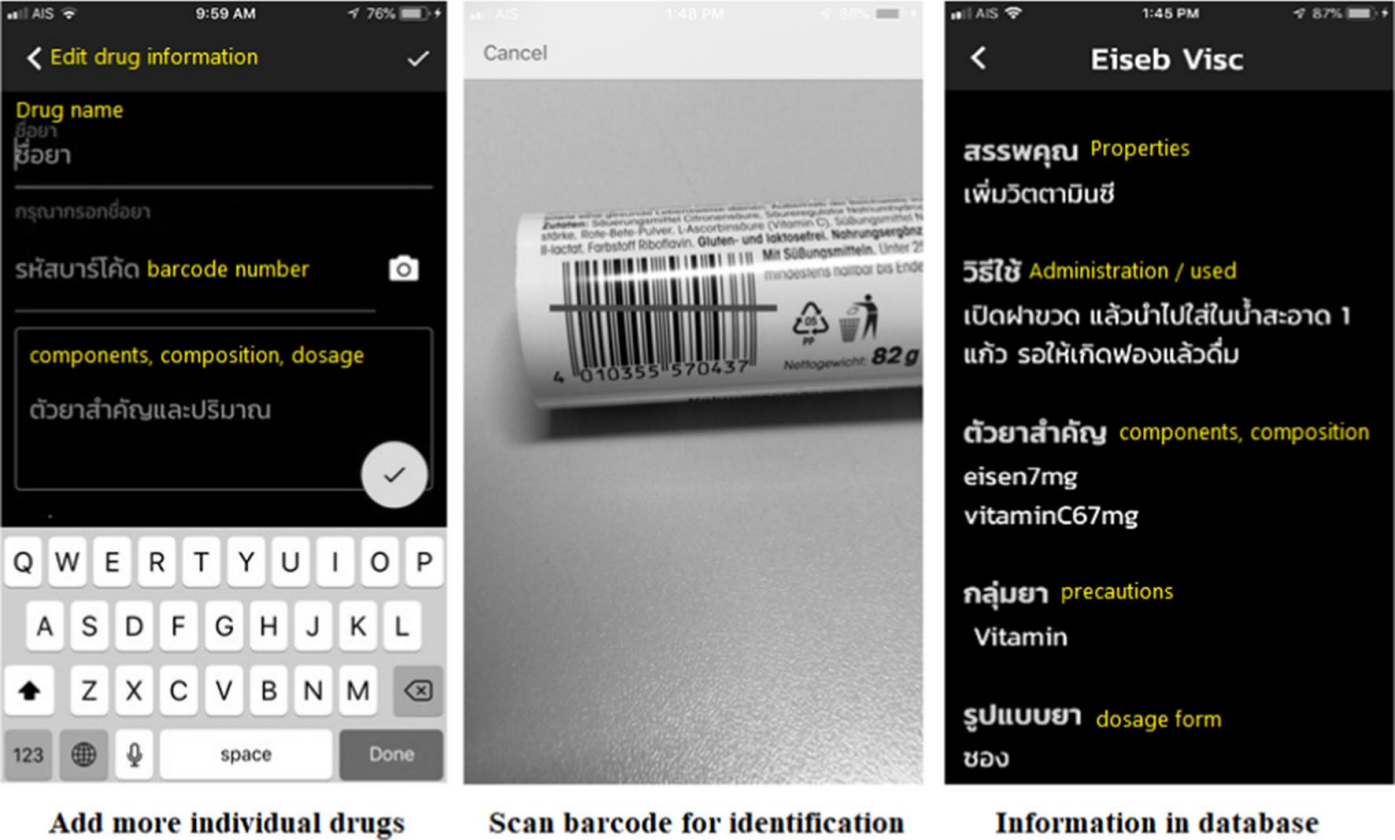
Medicinal database function. Purpose: For users with visual disabilities to create a personal medicinal database for future use

The answers to the research question of what an appropriate user interface design of a system for visually-impaired users should be indicated whether the elements on the screen were suitable for the use of people with visual disabilities based on considering the design of the user interface and each function of the application. The application design was created as a wireframe, which is an informal design based on an analysis of the functionalities and design, together with considering pain points and users’ problems. The functions of the application were analysed and designed as shown in Figs. 1, 2, 3, 4 and 5 and Table 5.

After finding the pain points of users’ problems, the user interface was created as a wireframe design to summarise all the elements on the screen, such as navigation components, input and output controls, screen proportion design, and menu list navigation to support drag and drop.

### Pharmaceutical content of application

The database contains monographs of 616 medicinal products available in Chiang Mai, including tropical medicine, wound-healing medicine, antiseptics, abdominal pain medication, laxatives, household medicines, non-dangerous drugs, vitamins, dietary supplements and cosmetics. Each of the monographs includes an indication of the active ingredient(s), dosage and administration, supply, storage and handling, side effects, drug interactions, as well as warnings and precautions. The information in the database was collected from reference books and the data of pharmaceutical companies was approved by Thailand’s Food and Drug Administration (FDA)**.**

### Results of application development

The Ru Tan Ya application was developed based on an iconic cross-platform framework to achieve the research objective. After evaluating the compatibility, errors, accuracy of content and quality of the proposed application, the final version contained five main functions. These included a function for searching for medicinal information, a medication adherence and timer function, a map function directing users to drug stores, a medication history function for users to record their individual medication history, and a function for users to create their own personal medicinal database.

The functional test was performed using iPhone 6 and Huawei P20 pro. One of the concerns at the time of the research was that the application must be able to operate on large screen devices due to the limited vision of visually-impaired users. Therefore, the analysis of requirements was based on considering the enhancement of visibility and the appropriation to the medical application in terms of practical usage [4].

**Fig. 6:**
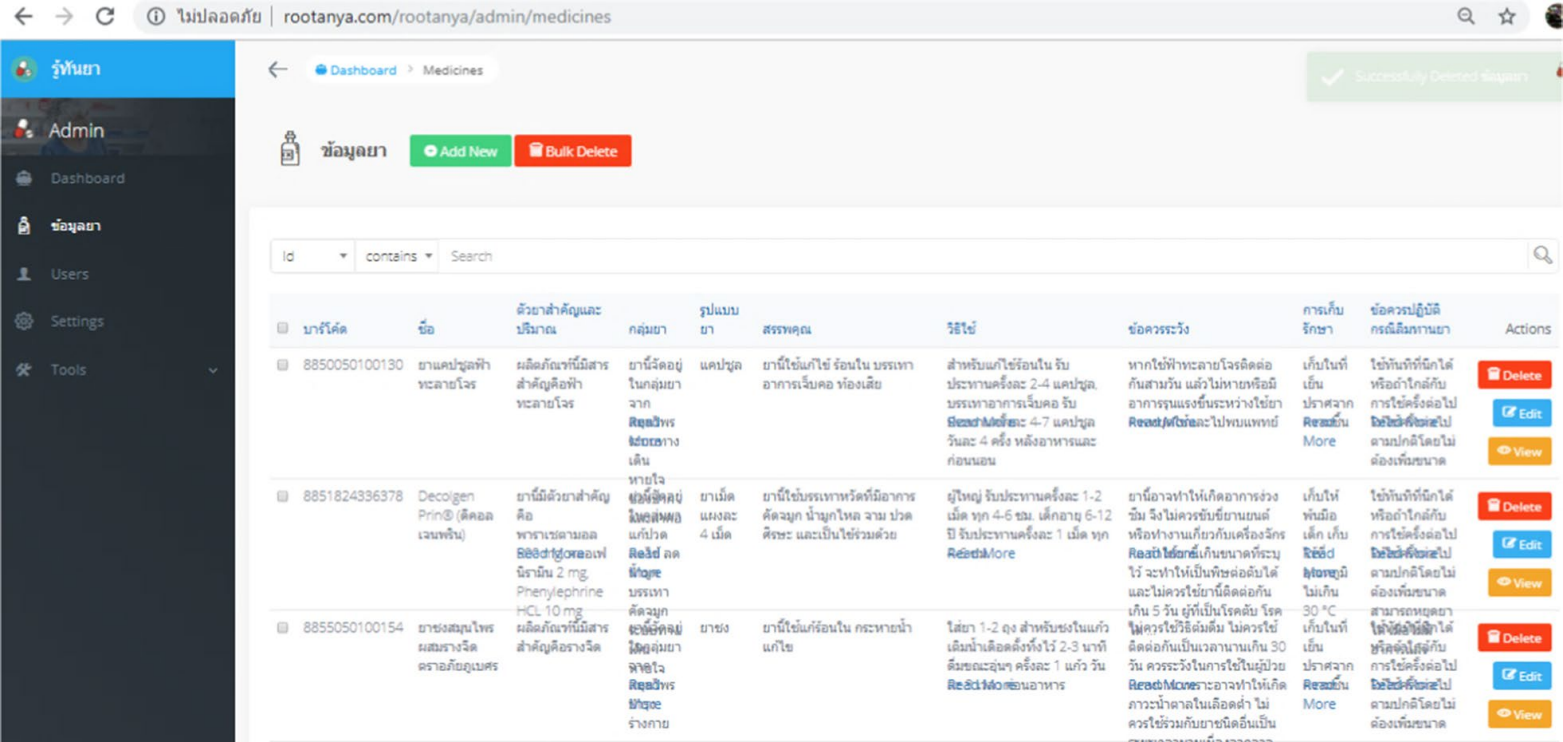
Snapshot of the back-end system of the application

**Fig. 7:**
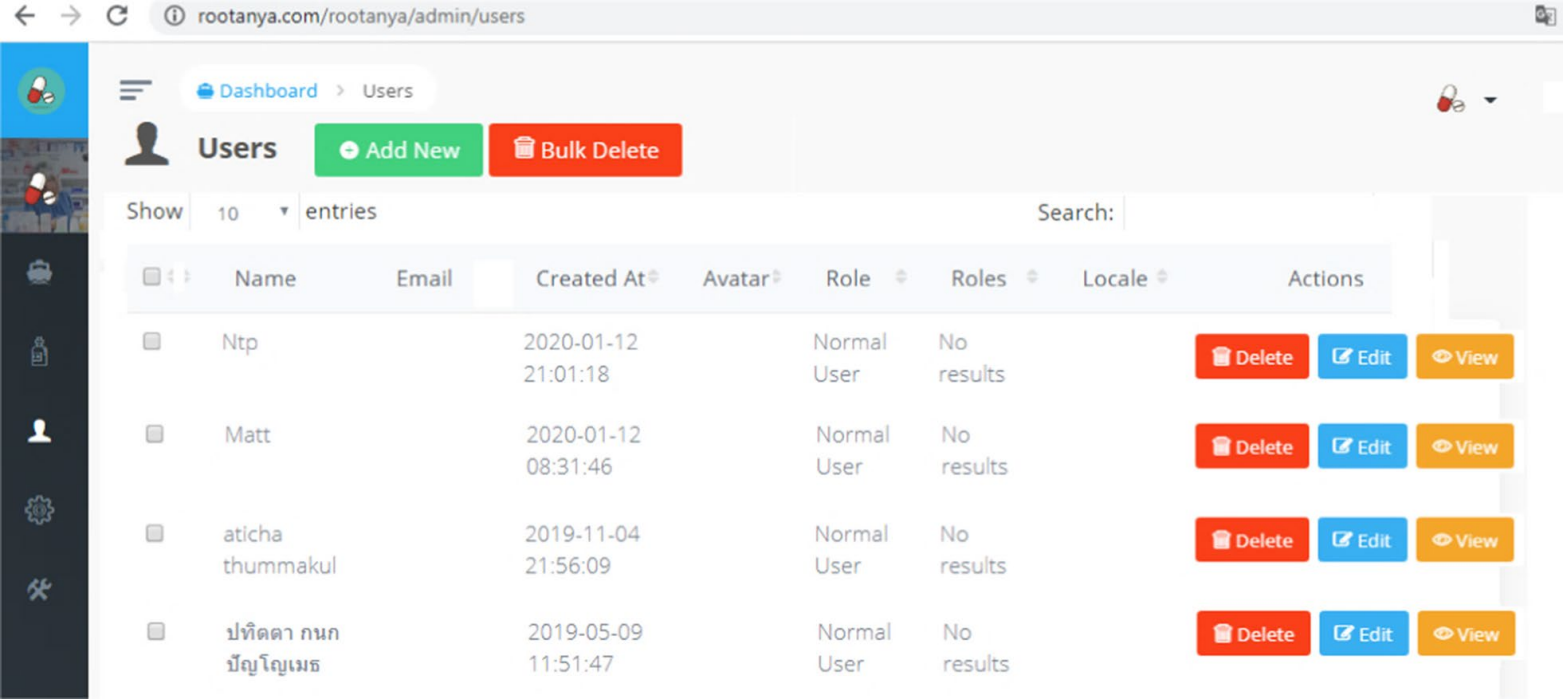
List of users who have already registered to use the Ru Tan Ya application

In addition, the back-end system of the Ru Tan Ya was also developed using Firebase to provide drug information and support the application. The database contained more than 616 medicinal product monographs. Figure 6 shows a snapshot of the back-end system of the application, while the list of users who have already registered to use the Ru Tan Ya application is shown in Fig. 7.

### Results of testing the application

#### Test of usability of functions

This step was taken to achieve the research objective of testing the usability of each function of the application and implementing it. The usability test was conducted to evaluate the design of the semi-final version of the application to ensure the quality and reliability of the proposed application. The frequency of the results of the scenario test, a cross-matrix usability test of each criterion (user motivation) compared to each function of the application, can be seen in Table 6.

**Table 6:** Results of cross-matrix usability test

		Point of consideration						
		Usability difficulties	Bug findings	Doubt on visual representation	Doubt on usage	Missing information	Misunderstandings	Total
Functional Test	1. Searching for medicinal information	10	3	3	4	2	12	34
	2. Medication adherence timer	15	12	8	4	2	10	51^(3)^
	3. In-app map direction of drug stores	13	16	5	6	2	12	54^(2)^
	4. Medication history function	14	13	9	3	4	4	47
	5. Medicinal database function	18	12	27	6	4	4	71^(1)^
	Total	70^(1)^	56^(2)^	52^(3)^	23	14	42	

(1) (2) (3) are rankings

As can be seen from the Table, the individual drug database function was rated top of the usability test results based on the samples’ evaluation of the use of the application (71 times). The majority of the samples expressed doubt about the visual representation (27 times), whereas usability difficulties were found 18 times. The map function was rated second-top of the test results (16 times due to bugs, 13 times due to usability difficulties and 12 times due to misunderstanding).

The medication adherence timer was rated third-top of the usability test results (15 times due to usability difficulties, 12 times due to bugs and 10 times due to misunderstanding). In addition, usability difficulties were found 70 times in the results of the overall usability (56 times due to bugs and 52 times due to doubt of visual representation).

#### User feedback

The second part of the test entailed the use of a questionnaire to evaluate users’ feedback.

**Fig. 8:**
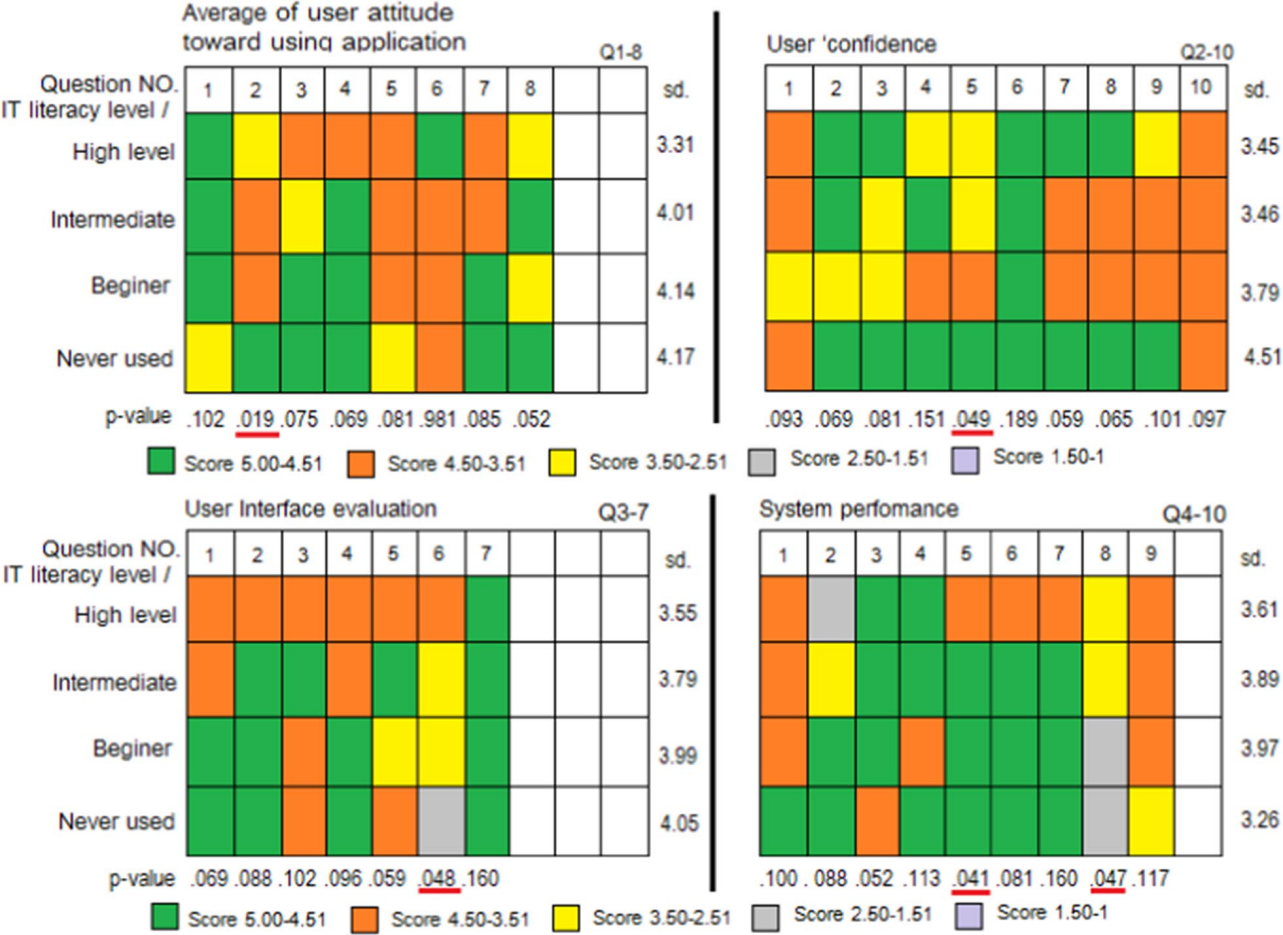
Application test based on users’ satisfaction and attitude toward using the application

Users’ satisfaction with the functionality of the application and their attitude toward using it were evaluated by interviewing the volunteers after completing the test of each function. The results of the evaluation, which correspond to the questions in Tables 7 and 8, are presented in Fig. 8.

**Table 7:** List of questions for users’ interview related to users’ attitude and users’ confidence

*Questions part 1 (Users’ attitude)*
Q1-1 Application could be a tool to provide information of primary care treatment rather than searching internet sites
Q1-2-Application would provide positive primary care treatment results
Q1-3-Application could provide precise information for healthcare treatment, comparable to professional advice from doctors or pharmacists
Q1-4-Application could help to improve self-healthcare
Q1-5-Application would change users’ behaviour and motivate them to practice self-healthcare
Q1-6-Application would not have adverse effects on users’ health.
Q1-7-Application would help users by reducing healthcare costs when illness occurs
Q1-8- Application would facilitate users’ primary treatment
*Questions part 2 (Users’ confidence)*
Q2-1 User is confident that the application works in providing advice for primary treatment
Q2-2 User is confident that the medicines information on the application is correct
Q2-3 User is confident that the application can provide precise information comparable to the advice of professionals, such as pharmacists
Q2-4 User is confident that the information on the application can help to understand how to use medicines and apply the information for immediate treatment
Q2-5 User is confident that the advice from the application is not harmful
Q2-6 User agrees to use the application and will recommend it to friends
Q2-7 User is confident that other people with visual disabilities can benefit from this application
Q2-8 User believes that the application can reduce healthcare costs when illness occurs
Q2-9 User believes that the application will facilitate users’ primary healthcare treatment more than searching for information via the internet
Q2-10 User is confident in downloading the application or recommending it to other visually-impaired people

**Table 8:** List of questions for users’ interview related to using the application in terms of user interface and system performance

*Questions part 3 *(*Usability Evaluation*)
Q3-1 Pressing buttons on the screen is easy and is supported by the slide style in the disability mode
Q3-2 Screen proportions are easy to access
Q3-3 The number of the menu items is suitable and easy to use
Q3-4 Each menu is easy to access and reasonably named
Q3-5 The proportion of the display areas is appropriate
Q3-6 The voiceover on the screen provides an easy way to communicate
Q3-7 The speed of the sound display is appropriate and easy to understand
*Questions part 4 (System Performance)*
Q4-1 The application is intuitive and understandable for users on the trial Q4-2 The application provides useful information about medication for primary self-treatment
Q4-3 The barcode scanner function makes it easy to find medicines
Q4-4 The time remaining function is useful for treatment
Q4-5 The search function using keywords or voice recognition makes searching easier
Q4-6 Information about historical drug consumption makes record-keeping easier
Q4-7 Information on the application enables users to self-manage their medication Q4-8 The individual drug database function is useful for recording medication information
Q4-9 The application can be operated quickly on a smartphone and the system is stable

The results were divided into four levels of user classification based on IT literacy skills to evaluate the correlation between the groups. Based on the average scores, the majority of the users, beginners and never-used groups, indicated that they were satisfied with the application in every evaluation section.

In part one, the majority of the participants agreed that the application could facilitate better self-healthcare and be a more efficient tool to search for primary-care treatment information. The application could provide appropriate primary healthcare treatment without adverse effects on users’ health. Based on these users’ attitude, the application was positively encouraging, since the results of Q1-3 indicated that they believed that the application could provide precise healthcare information comparable to professional advice from doctors or pharmacists. However, statistically significant differences were found in question 2, which was about the positive primary care treatment from the system. These almost statistically significant differences (p < 0.05) demonstrated that majorities in all groups did not agree with this statement.

Among the questions designed to determine users’ confidence in using the application, the answers to Q2-10 showed that they were confident that the application would not harm them and could provide precise information about drugs. They believed that the application could reduce the cost of healthcare at times of illness. Hence, users’ confidence in the application reflected their belief in its benefits. There were no significant statistical differences in the scores of the usability evaluation of the other questions, except Q5, where the p-value was 0.049. The comparison results showed that each group of volunteer users did not agree with the statement about their confidence in taking advice from the application because it was not harmful.

Questions Q3-7 were designed to evaluate the user interface. The element with the highest score was the press-button function on the screen, since it was supported by the slide-rule style in the disability mode and easy to use. The volunteers found that screen proportion was easy to access, each menu was easy to navigate, and the sound display was at an optimum speed and easy to understand. Therefore, the compatibility of touching the screen, the proportion of the screen, the sound display and the menu elements were found to have a positive impact on users. As for the comparison result in each group, there were no significant statistical differences except Q6 which was about whether the voiceover on the screen could provide an easy way to communicate. The results also showed that the average score of the never-used and beginners groups were lower than those of the other groups, whereas the comparision of each group showed a significant statistical difference of 0.048 (p < 0.05).

In terms of users’ confidence in using the application, Q4-9 were designed to evaluate the system’s performance, and the results indicated that the application was able to provide useful information about medication and medication adherence, as well as possessing a timer function. Furthermore, useful information could be acquired from the application, which can quickly operate on a smartphone, and the system is stable and performs well. Therefore, this application tends to be beneficial for users in inputting missing information and offering some essential medical-related services. The results of the volunteer users’ feedback scores for Q5 which asked about the search function by using keywords or voice recognition for easier searching, were different for each group with a p-value of 0.041. Hence, users in each group of volunteers did not agree with this statement, especially the never-used group, which had the lowest score of all the groups.

Moreover, the results of Q8, which asked if the individual drug database function was useful for recording medication information, were different for each group with a p-value of 0.047. This showed that each group did not agree that this function was useful, especially the never-used and beginner groups, whose score was lower than that of the other groups.

#### Long-term monitoring and investigation results

**Fig. 9:**
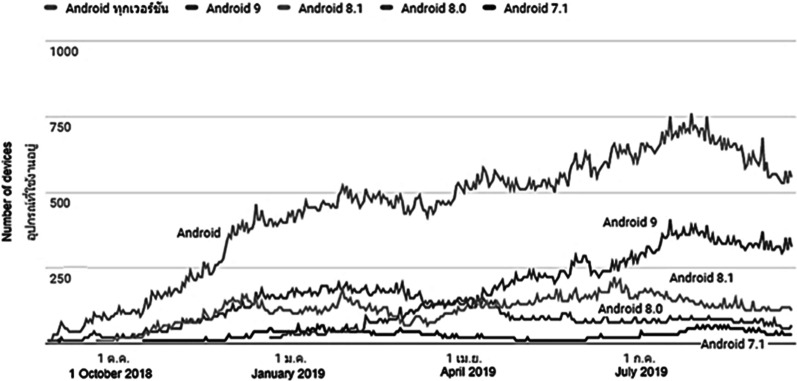
Application download statistics reported by Google Play in 2019

The information provided in this section supports the Research Policy and Strategy to identify the key performance indicator of the internal research achievement of the College of Arts, Media and Technology (CAMT), Chiang Mai University (CMU). The results were derived from the Google developer account, “CAMT” which was accessed via Google.google.com/apps/ publish, where the proposed application is shared publicly. As for the long-term monitoring and investigation, the research was completed in October, 2018 and the results were reviewed from October, 2018 to July, 2019. The application was downloaded approximately 750 times during the 9 months of the investigation. It can be seen from Fig. 9 that the trend of downloading the app increased from October, 2018 and then decreased from July, 2019. However, this was a small number of downloads compared to the entire visually-impaired population of Thailand, since the target experimental group was based in Chiang Mai and the proposed application was promoted directly to them. In this case, it can be assumed that referrals among users can be expected if the application performs well. However, the application was promoted on both iOS and android platforms, as shown in Fig. 9, and the reported number of iOS users could not be verified due to technical problems and Apple’s company policy. Note that the information from the Fig. 9 was downloaded from the play stroe console developers since 2019.

**Fig. 10:**
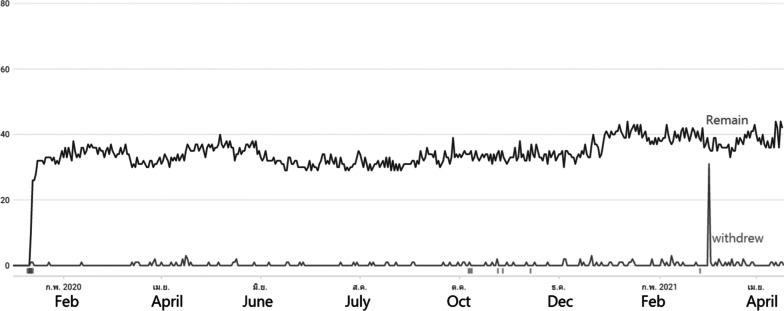
Application download statistics reported by Google Play for specified group (visually-impaired participants n = 60)

In 2021, the application was uploaded into the new account after being revised and updated due to the Google Play policy. The reported number of remaining application users was 198. While 44 of the 60 participants were still using the application, as shown in Fig. 10, the others had stopped using it because their smartphones could not update the information from Google Play console developers due to their version of android.

## Discussion

The the main problem from this studies that it was difficult to use smart technology based on their experience due to functionality issues, difficulty in accessing information from various sources, misunderstanding the information provided by the system and the fact that most applications did not support a disability mode [11, 17–19]. This showed that the main problem with currently-available mobile pharmaceutical applications or any mobile applications [16] is a lack of development for visually-impaired users.

The purpose of this study was to design a mobile application with an appropriate user interface and functionality to increase the usability for visually-impaired users in Thailand and enable them to access primary self-treatment when they are sick. The application was designed and analysed based on a user-centred design process [26, 39], which included five stages, as well as considering users’ needs and the concept of designing a user-interface, usability, and accessibility [31–36]. the final version of the application was released to sixty visually-impaired volunteers, who had agreed to test it.

It was found from the results of a cross-matrix usability test that most of the low usability of the function was associated with receiving input from users, such as typing a word or finding a textbox to input a word. This problem occurred in the Medicinal database function, which was the first order. This case was supported by the results of the questions for users’ interview related to users’ attitude and users’ confidence, which confirmed that it was difficult to use this function.

In addition, it was found that positioning elements, such as some of the buttons, did not immediately respond (more than 5 s) due to cloud-based information retrieval. As a result, users were unsure if the system could respond or was unable to do so. Hence, the functions that involved input, such as the medicinal database function, In-app map direction of drug stores and medication adherence timer were the top considerations of the output, respectively.

To answer the research question, some functions of this application that require users to input information may not be suitable for visually-impaired users, and the voiceover and voice recognition functions were also problematic due to pronunciation, the requirement of IT skills and the need to redesign the user interface [20]. These problems were supported by the result of users’ interviews in relation to the user interface and system performance of the application usage.

In the users’ level of satisfaction evaluation with the application and their attitude toward using it were tested and the results showed that users with different levels of proficiency had different levels of satisfaction [41]. Highly proficient users and those in the intermediate group were more satisfied than beginners who had never used such an application, and the highly proficient group also had a better attitude toward using the application, the user interface and the system’s performance. These results from users’ feedback demonstrated that users’ IT literacy and usage background are key to developing a successful application [20, 22, 40].

Therefore, visually-impaired users are encouraged to fully use smart devices based on their level of IT literacy and their background. If they have basic skills, such as information literacy, searching for data through a smart device and developing a smart system may fail to assist them. To improve the application’s performance after the first development based on evidence from the the research, if users are not skilled in using a smart device, the application will be used by a relative or with the assistance of a pharmacist or family member.

Consequently, the application proposed for development in this study is suitable for the use of visually-impaired people and the steps involved in developing it were shown in detail [33]. The outcome of this research is expected to assist visually-impaired people in Thailand [14, 15], and it may be considered to be the first step in the development of smart technology (to TRL 3) to achieve social equality and improve the quality of life of people with disabilities.

## Conclusion

The steps involved in designing and developing a mobile pharmaceutical application for visually-impaired groups based on analysing users’ problems were demonstrated in this study. It was found from testing the application that some functions may not be suitable for visually-impaired users, such as those that require users to input information. The voiceover and voice recognition functions were also found to be problematic due to pronunciation and the need of IT skills. This issue indicates that users’ ability to use the application depends on their background, IT skills and experience of smart technology.

While the application may have a high functional capacity, the gap in healthcare services between the general public and disabled groups will still exist if users have inadequate IT skills. Moreover, it was found that some functions, such as the personal medicine database, may be suitable for use with the aid of pharmacists or other sighted individuals rather than visually-impaired users themselves. too many fields are difficult to input, despite the use of voiceover; therefore, functions such as the input of data via voice need to be reconsidered and redesigned.

Therefore, this function may be more suitable for general users because it can assist them to record information about the medicines being purchased at the time. It is also convenient for users who need to self-medicate. Although the application was uploaded to the application store, and the number of downloads is increasing, the design of some functions needs to be improved in the next version.

### Limitations

This research has several notable limitations, the first of which is that it was based on a single group of end-users in Chiang Mai province consisting of visually-impaired people who are smartphone users. This was quite a small group of participants who had been educated to different academic levels. This may have affected the results of the usability test. Additionally, the application designed in this study contained basic medication for a specific condition limited to the monographs of 616 medicinal products.

In terms of the application design, some limitations were caused by requiring users to input information. Therefore, a follow-up design should focus on increasing the voiceover and voice recognition functions to make it convenient for visually-impaired users to use the application. Moreover, a redesign of the user interface to increase usability is strongly recommended to improve the application’s performance.

### Availability and requirements

- Project name: Mobile application development for medications managing for people with vision disabilities and elderly patients
- Project home page: https://play.google.com/store/apps/details?id=medical.camt.cmu.ac.th.medicalapp
- Operating system(s): Android
- Programming language: Java
- Other requirements: Java 1.3.1 or higher
- License: freely available
- Any restrictions to use by non-academics: not restrictions

## Supplementary Information

**Additional file 1.** Human-computer interaction (HCI) guidelines.

## Data Availability

The datasets used and/or analysed during the current study are available from the corresponding author on reasonable request.

## Abbreviations

TRL: Technology. Readiness levels
IT: Information technology
HCI: Human computer interaction
FDA: Thailand’s Food and Drug Administration
