# Supplementary material for: Pharmaceutical mobile application for visually-impaired people in Thailand: development and implementation
Source: BMC Med Inform Decis Mak. 2021 Jul 16;21:217. doi: 10.1186/s12911-021-01573-z (PMC8283832; doi:10.1186/s12911-021-01573-z)
Supplement: Supplementary file 1 — Additional file 1. Human-computer interaction (HCI) guidelines. [file 12911_2021_1573_MOESM1_ESM.docx]

**Supplement 1**

**Human-computer interaction (HCI) guidelines.**

| **Eight Golden Rules [39]** | **Norman’s Seven Principles [38]** | **Nielsen's Ten Heuristic**  **[20]** |
| --- | --- | --- |
| 1. Strive for consistency | 1. Mapping: Use knowledge both in the world & in the head | 1. Visibility of system status. |
| 2. Cater for universal usability | 2. Simplify task structures | 2. Match system and real world |
| 3. Offer informative feedback | 3. Visibility: Make things visible | 3. User control and freedom |
| 4. Design dialogue to yield closure | 4. Get the mapping right (User mental model = Conceptual model = Designed model) | 4. Consistency and standards |
| 5. Prevent errors | 5. Convert constraints into advantages (Physical, Cultural, and Technological constraints) | 5. Error prevention |
| 6. Permit easy reversal of actions | 6. Design for Error | 6. Recognition rather than Recall |
| 7. Support internal locus of control | 7. When all else fails, Standardize | 7. Flexibility and efficiency of use |
| 8. Reduce short-term memory load |  | 8. Aesthetic and minimalist design |
|  |  | 9. Helps diagnosis and recovery from errors |
|  |  | 10. Documentation and Help |
